# Temporomandibular joint reconstruction with alloplastic prosthesis: the outcomes of four cases

**DOI:** 10.1186/s40902-017-0103-7

**Published:** 2017-03-25

**Authors:** Jung-Hyun Park, Eun Jo, Hoon Cho, Hyung Jun Kim

**Affiliations:** 1 Department of Oral and Maxillofacial Surgery, College of Dentistry, Yonsei University, 50 Yonsei-ro, Seodaemun-gu, Seoul, 120-752 Republic of Korea; 2 Present address: Department of Oral and Maxillofacial Surgery, Ewha Womans University, Seoul, Republic of Korea

**Keywords:** Temporomandibular joint, Reconstruction, Alloplastic prosthesis, Biomet, Indication

## Abstract

**Background:**

The purpose of this study is to evaluate the outcomes of four patients receiving stock Biomet TMJ prosthesis for reconstruction of the TMJs.

**Methods:**

TMJ reconstruction with stock Biomet TMJ prosthesis was performed in four patients who had joint damages by trauma, tumor, resorption, and ankylosis, which represent the indications of alloplastic prosthesis.

**Results:**

Loss of condyle from trauma and resorption of joint are good indications for prosthesis, but the patients should be informed about limitation of jaw movement. In case of structural damage of TMJ by tumor, tumor recurrence should be considered before planning TMJ reconstruction. Considering heterotopic bone formation in case of ankylosis, periodic follow-up and special surgical technique are required.

**Conclusions:**

Given careful treatment planning and understanding the functional limitation of TMJ prosthesis, alloplastic prosthesis is a safe and effective management option for the reconstruction of TMJs.

## Background

Anatomic structural damages of temporomandibular joints (TMJs) such as trauma, tumor, resorption, and ankylosis require removal of pathologic structures and reconstruction of TMJs. The goals of TMJ reconstruction are (1) improved mandibular form and function, (2) reduction of pain and disability, (3) containment of excessive treatment and cost, and (4) prevention of further morbidity [1]. Alloplastic total TMJ reconstruction is a management option for patients with anatomically and pathologically compromised dysfunctional TMJs. Several devices are available, including TMJ Concepts (Ventura, CA, USA), TMJ Implants (Golden, CO, USA), and Biomet (Jacksonville, FL, USA). In contrast to an individually designed prosthesis, such as TMJ Concepts, Biomet is a stock product system. It is a two-component system comprised of fossa and mandibular components which are available in several sizes. Using templates, surgeons can select suitably sized components during the operation. The stock prosthesis has advantages of lower cost, shorter treatment time frames, and more placement versatility than customized prosthesis [2]. Recently, as the use of stock alloplastic TMJ prosthesis (Biomet) has increased, several studies have reported stable and satisfactory results [3–5]. The authors present the outcomes of four patients receiving TMJ reconstruction using Biomet involving, respectively, trauma, tumor, resorption, and ankylosis.

## Case presentation

### Case 1

**Fig. 1:**
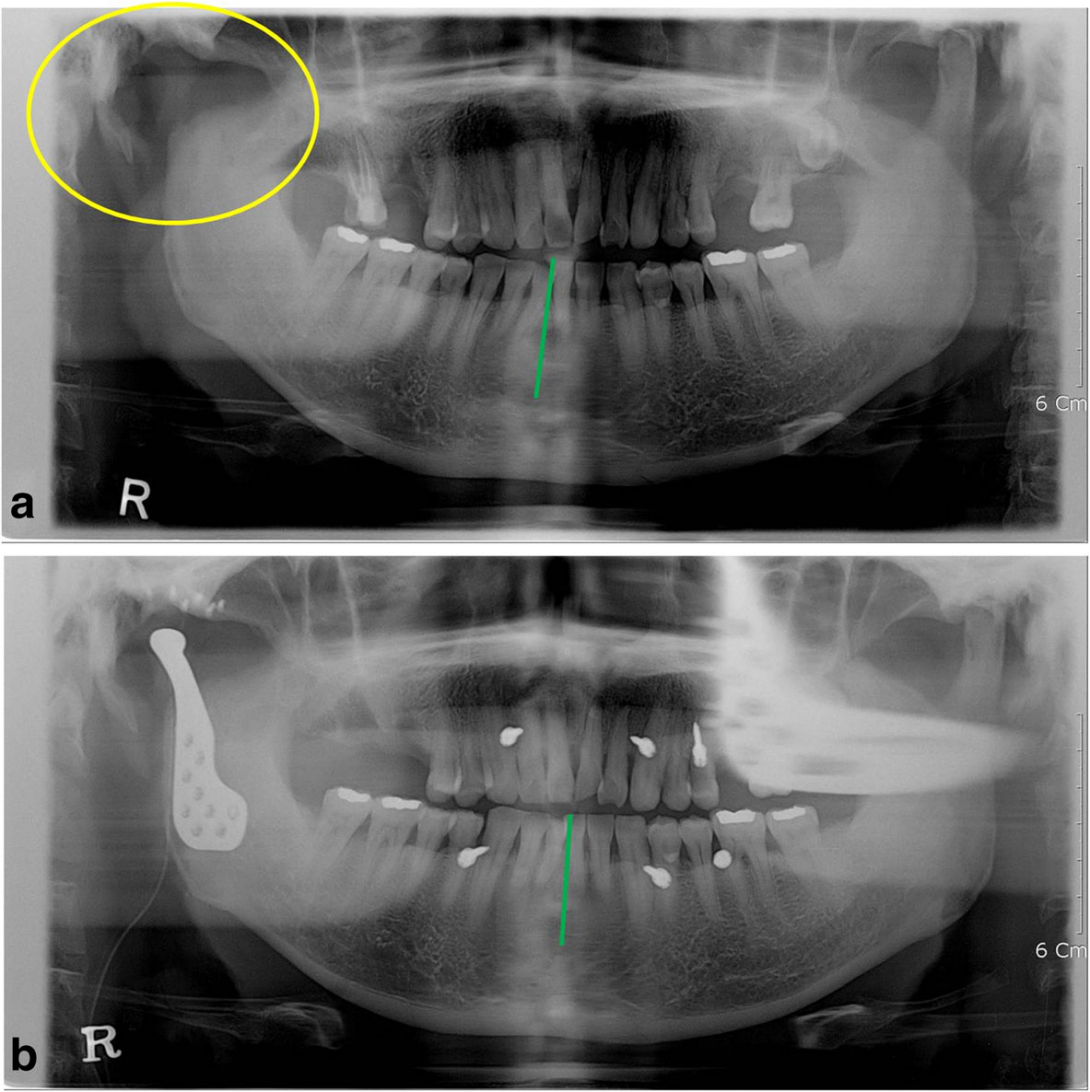
Case 1. **a** The mandible was deviated to the right (*green line*) as a consequence of condylectomy (*yellow circle*). **b** The panoramic radiography taken 1 day after the operation showed recovery of original occlusion (*green line*). Intermaxillary fixation screws were used to secure occlusion during operation

A 52-year-old man who had suffered from malocclusion after condylectomy presented. He had fallen down the stairs and broken his mandible, right condylar neck. He had had a surgical intervention, open reduction with fixation, in another hospital. After the operation, the right TMJ area had become infected, so condylectomy had been performed. One month after condylectomy, he presented to Yonsei University Dental Hospital. His mandible was deviated to the right at rest, and occlusion was unfavorable as a consequence of condylectomy (Fig. 1a). The range of mouth opening was limited to 30 mm of maximum interincisal distance. After infection control using oral antibiotics, reconstruction of the right TMJ with a joint prosthesis was planned. During the operation, surgeons secured stable occlusion by intermaxillary fixation to restore original occlusion. Installation of the TMJ prosthesis was carried by two incisions: preauricular and Risdon’s. The panoramic radiography taken 1 day after the operation showed recovery of original occlusion (Fig. 1b). He was discharged after 3 days without postoperative complications. He has been followed up for 1 year with no problem from prosthesis having developed. The prosthesis has functioned well, and his occlusion has been stable, although the maximum interincisal opening is still 30 mm.

### Case 2

**Fig. 2:**
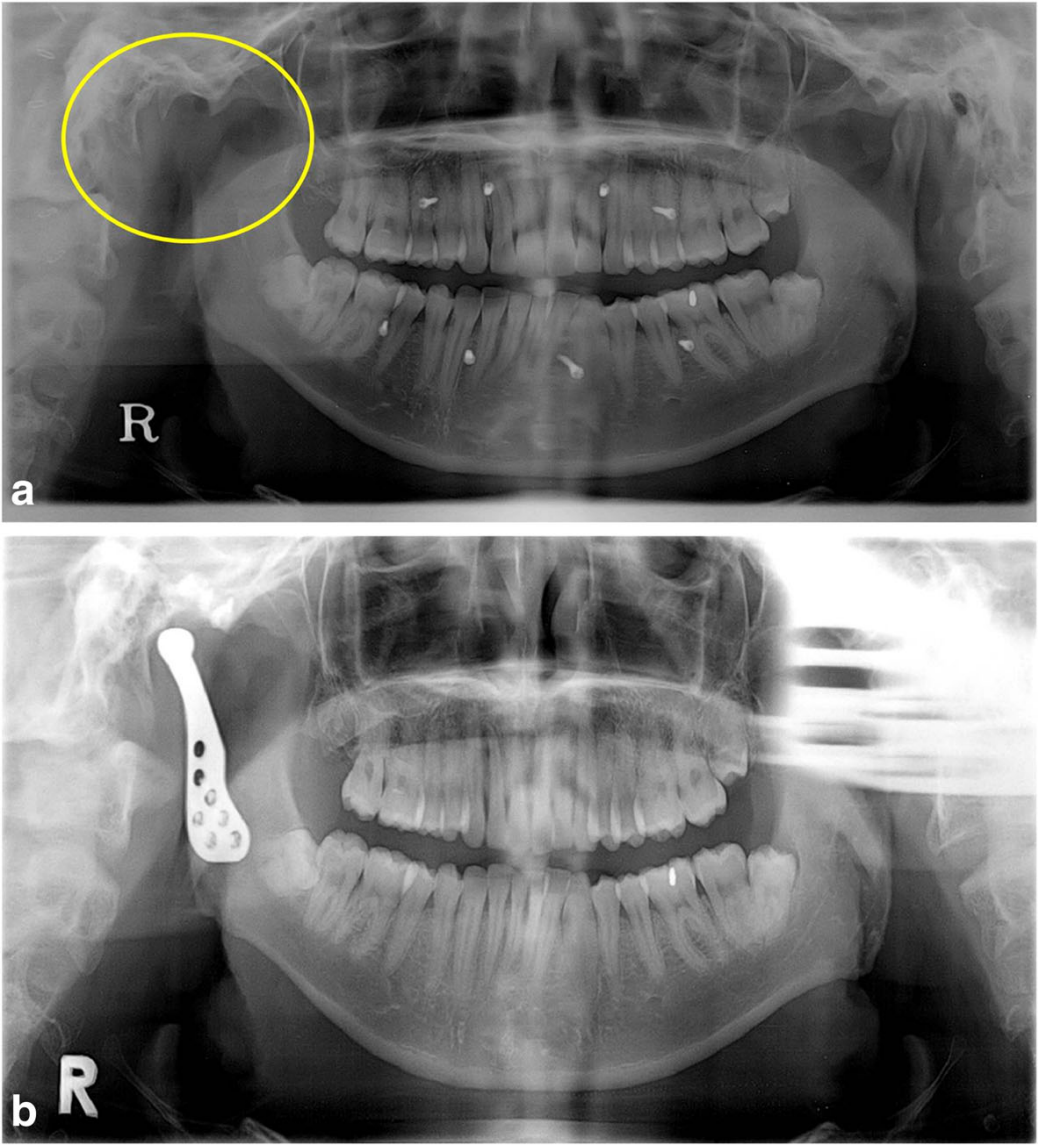
Case 2. **a** Right condyle was removed (*yellow circle*) because of carcinoma of the external auditory canal. Intermaxillary fixation screws and elastic bands were used to maintain his occlusion for 8 months. **b** At 1 month after the TMJ reconstruction, intermaxillary fixation screws were removed and stable occlusion was confirmed

A 34-year-old man was diagnosed with adenoid cystic carcinoma of right external auditory canal. He underwent an operation to remove carcinoma with right condylectomy in the Department of Otolaryngology (Fig. 2a). After the operation, his occlusion was maintained by intermaxillary fixation screws and elastic bands. He had no limitation of jaw opening, but his mandible was deviated to the right side when jaw opened. After 8 months of first operation, reconstruction of right TMJ was planned to maintain stable occlusion without intermaxillary fixation screws. During the operation for TMJ reconstruction, there was no abnormal finding around the previous operation site. After TMJ reconstruction, intermaxillary fixation screws were removed and stable occlusion was confirmed (Fig. 2b). Three months later, however, sharp pain and mild swelling developed in the right TMJ area. The symptom persisted in spite of using antibiotics and NSAIDs. Computed tomography (CT) was taken but no abnormality could be found because of metal artifacts from the prosthesis. After four more months, magnetic resonance imaging (MRI) revealed re-growing tumor along the temporalis muscle. Additional surgeries were performed to remove tumor and prosthesis, but he is still suffering from uncontrolled primary tumor in the right TMJ and temporal area.

### Case 3

**Fig. 3:**
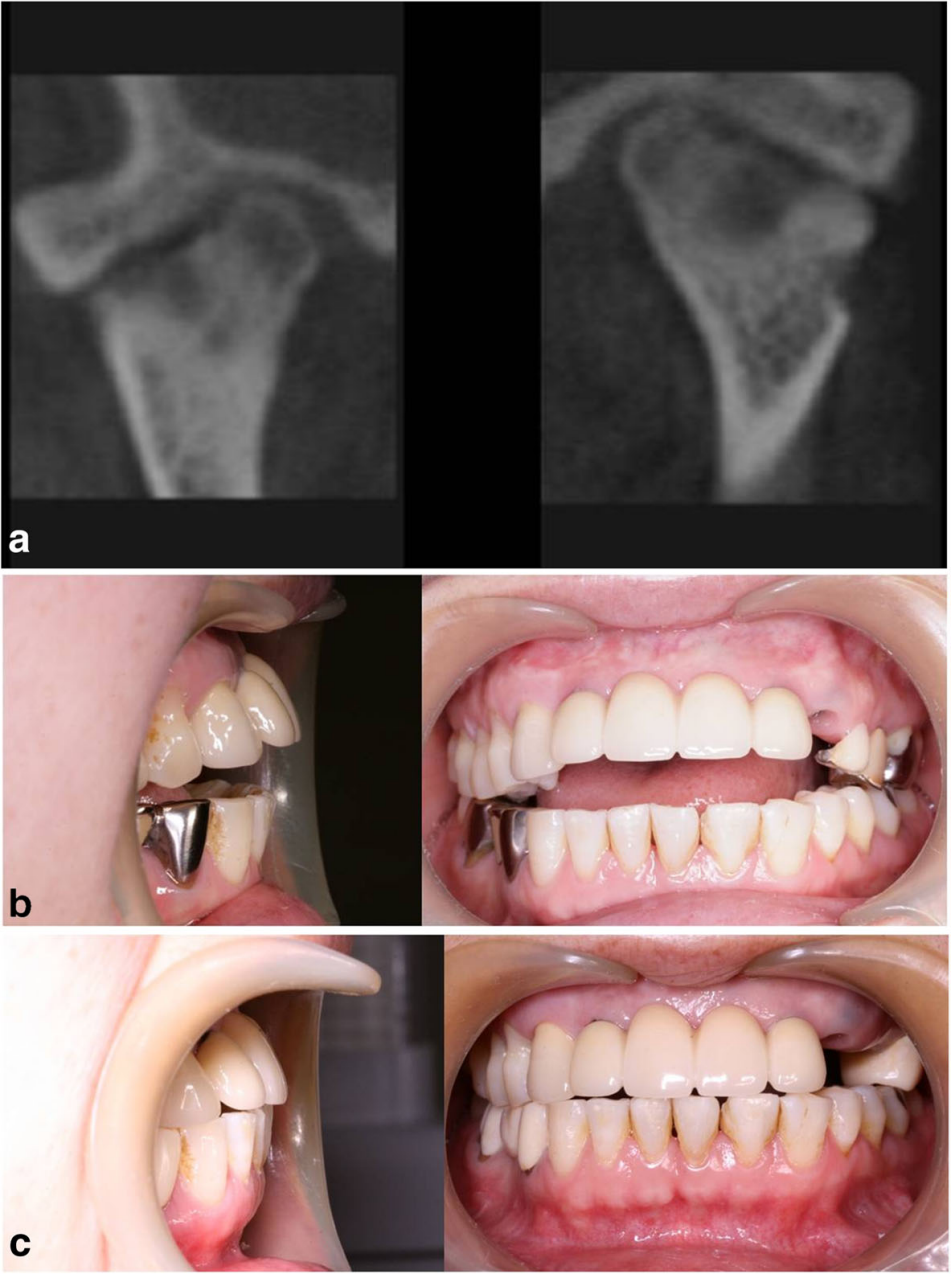
Case 3. **a** CT images showing flattening of bilateral condylar heads, irregularity of articular surface, and narrowing of joint spaces. **b** Anterior open bite developed with overbite changing from 0 to −6 mm during splint therapy. **c** Anterior open bite was closed, and no further progression of anterior open bite has been observed

A 53-year-old woman with rheumatoid arthritis of the hand, wrist, and shoulder presented by reason of pain on both TMJs. She had suffered from rheumatoid arthritis for 1 year and had been taking anti-inflammatory drugs. CT confirmed that both TMJs were also affected by rheumatoid arthritis (Fig. 3a). Limited mouth opening (maximum interincisal distance of 23 mm) was observed, and pain was aggravated when she moved her jaw. For pain relief, dental splint therapy was started and arthrocentesis of both TMJs was performed. The symptom improved with the treatments, but anterior open bite developed slowly. Overbite changed to −6 mm from initial overbite of 0 mm (Fig. 3b). To prevent disease progression, a reconstruction of TMJs with prosthesis was planned. During the operation, superior repositioning of posterior maxilla using Le Fort I osteotomy was performed because her upper anterior teeth protruded. After resection of both condyles, the reconstruction of TMJs with prosthesis was done with counterclockwise rotation of mandible to close anterior open bite (Fig. 3c). It has been 2 years since the operation, and there has been no evidence of any persisting inflammatory process. Neither further progression of anterior open bite nor symptoms of inflammation have been observed.

### Case 4

**Fig. 4:**
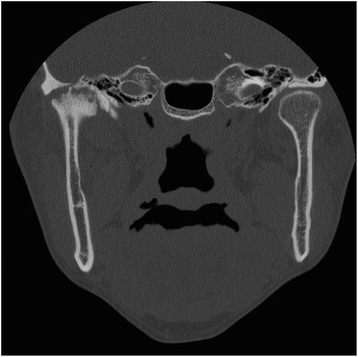
Case 4. Coronal CT image shows ankylosis of the right TMJ

A 41-year-old man who had suffered from ankylosing spondylitis since the age of 31 years presented. He was complaining of pain in the right TMJ area and limitation of mouth opening. He could not open his mouth more than 25 mm, and his jaw deviated to the right side which meant limitation of right TMJ movement. By examination of CT images, ankylosis of right TMJ secondary to ankylosing spondylitis was suspected (Fig. 4). Reconstruction of right TMJ with prosthesis was planned to eliminate the possibility of re-ankylosis of TMJ. During the operation, a fibrous mass and the ankylosed condyle were removed, the glenoid fossa was trimmed, and a TMJ prosthesis was installed. Three months later, his maximum interincisal distance improved to 36 mm but his jaw still deviated to the right side. Neither inflammatory symptom nor heterotopic bone formation of the right TMJ has been observed for 1 year. He is under periodic follow-up because ankylosis could recur around joint prosthesis and the left (non-affected side) TMJ could also be involved by ankylosis secondary to ankylosing spondylitis.

## Discussion

TMJ reconstruction with alloplastic prosthesis is indicated in cases of specific TMJ conditions and pathology with irreversible joint damage [6]. The authors described the outcomes of four patients receiving TMJ reconstruction using stock prosthesis involving, respectively, trauma, tumor, resorption, and ankylosis (Table 1). In case 1, the patient had experienced trauma, had fixative surgery, contracted a postoperative infection, and then undergone a condylectomy. Before installation of the prosthesis, it was important to control all infection, which could cause reconstruction failure. As an infected prosthesis can lead to secondary operation and removal of the prosthesis [7, 8], control of previous infection is important before its installation. The chief complaint of the patient was malocclusion after loss of the condyle. After reconstruction with a TMJ prosthesis, original occlusion was restored although without former range of mouth opening. An infection which had persisted over a month and multiple surgeries are considered a reason for limitation of mouth opening.

**Table 1:** Four patients receiving TMJ reconstruction using alloplastic prosthesis

	Case 1	Case 2	Case 3	Case 4
Age/gender	52/male	34/male	53/female	41/male
Joints	Right	Right	Bilateral	Right
Etiology	Trauma	Tumor (carcinoma of right external auditory canal)	Rheumatoid arthritis	Ankylosing spondylitis
Preoperative symptom	Deviated mandible, unstable occlusion	Deviated mandible	Anterior open bite	Pain, limited mouth opening
Postoperative symptom	Limited mouth opening	Sharp pain and swelling by tumor recurrence	No further inflammation	Mandibular deflection when opening the mouth
Follow-up periods	1 year	7 months	2 years	1 year

In case 2, involvement of the TMJ by carcinoma of the external auditory canal necessitated condyle resection. Although reconstructive surgery was successful, the patient still suffered from uncontrolled primary tumor. When the symptoms of tumor recurrence first developed, they were ascribed to postoperative infection of prosthesis because metal artifacts from the prosthesis made the images difficult to interpret. Alloplastic prosthesis is a reconstructive option for tumors around TMJs but is not suitable to patients who need to have a CT or MRI taken to identify further pathologic changes.

Severe inflammatory joint disease is another indication for alloplastic TMJ prosthesis. In case 3, severe inflammation of TMJs caused bilateral condylar resorption in rheumatoid arthritis. Alloplastic reconstruction of TMJ was planned to stop disease progression, which was aggravating anterior open bite. Severe inflammatory joint disease has been reported to have the best results with alloplastic reconstruction in terms of predictable results [1]. Counterclockwise rotation of mandible is considered a destabilizing factor in orthognathic surgery. Using a prosthesis, however, stable function without relapse is expected according to the literature [6, 9]. The patient has been followed up for 2 years with no evidence of relapse.

TMJ ankylosis is also a good indication for reconstruction with prosthesis, especially in patients with recurrent fibrosis and bony ankylosis [10]. In case 4, the risk of re-ankylosis after gap arthroplasty was considered high because of the underlying disease, ankylosing spondylitis. Total reconstruction of TMJ with prosthesis was planned to avoid the risk of re-ankylosis. The patient has restored his former range of mouth opening, but his jaw still deviates to the affected side, which is accounted for by the prosthesis rather than re-ankylosis. In contrast with TMJ, which functions in both rotational and translational patterns, the prosthesis functions in a purely rotational pattern due to the loss of lateral pterygoid muscle attachment. Limited movement of jaw to the nonprosthesis side and deviation of jaw to the prosthesis side are inevitable.

There are some limitations of TMJ reconstruction with alloplastic prosthesis. Because alloplastic prosthesis cannot follow growth, the use of prosthesis in a growing patient is limited. Long-term data on material wear and stability of TMJ prosthetics is still needed. Nevertheless, due to its advantages over autogenous graft such as (1) immediate jaw function, (2) low risk of re-ankylosis, (3) no need for a secondary donor site, (4) decreased surgery time, and (5) mimicking normal anatomy [1], alloplastic prosthesis is widely used to reconstruct TMJs.

Several studies have reported satisfactory results of TMJ reconstruction with alloplastic prosthesis. Mercuri et al. reported long-term outcome of 193 patients (mean follow-up of 11.4 years), which showed a significant reduction in pain and an increase in mandibular function and range of motion after TMJ reconstruction using customized prosthesis (TMJ Concepts) [11]. A study by Westermark who evaluated 12 patients treated with stock prosthesis (Biomet) after a follow-up time of up to 8 years reported an increased mean jaw-opening capacity and elimination of joint-related pain [12]. Also, a 3-year follow-up study of 288 patients treated with stock prosthesis (Biomet) by Giannakopoulos et al. showed significant improvement in pain level, jaw function, and incisal opening [13].

The above four cases of trauma, tumor, resorption, and ankylosis represent the indications of alloplastic prosthesis. Loss of condyle from trauma and resorption of joint are good indications for prosthesis, but the patients should be informed about limitation of jaw movement. In case of structural damage of TMJ by tumor, recurrence of tumor should be considered before planning TMJ reconstruction. Considering heterotopic bone formation in case of ankylosis, periodic follow-up and special surgical technique are required.

## Conclusions

TMJ reconstruction with alloplastic prosthesis is indicated in cases of specific TMJ conditions and pathology with irreversible joint damage. Given careful treatment planning and understanding the functional limitation of TMJ prosthesis, alloplastic prosthesis is a safe and effective management option for the reconstruction of TMJs.

## References

[1] Mercuri LG (2000). The use of alloplastic prostheses for temporomandibular joint reconstruction. J Oral Maxillofac Surg.

[2] Gonzalez-Perez LM, Gonzalez-Perez-Somarriba B, Centeno G, Vallellano C, Montes-Carmona JF (2016). Evaluation of total alloplastic temporo-mandibular joint replacement with two different types of prostheses: a three-year prospective study. Med Oral Patol Oral Cir Bucal.

[3] Roh YC, Lee ST, Geum DH, Chung IK, Shin SH (2013) Treatment of Temporomandibular Joint Disorder by Alloplastic Total Temporomandibular Joint Replacement. Journal of Korean Association of Maxillofacial Plastic and Reconstructive Surgeons 35:412–20

[4] Leandro LF, Ono HY, Loureiro CC, Marinho K, Guevara HA (2013). A ten-year experience and follow-up of three hundred patients fitted with the Biomet/Lorenz microfixation TMJ replacement system. Int J Oral Maxillofac Surg.

[5] Westermark A (2010). Total reconstruction of the temporomandibular joint. Up to 8 years of follow-up of patients treated with Biomet((R)) total joint prostheses. Int J Oral Maxillofac Surg.

[6] Dela Coleta KE, Wolford LM, Goncalves JR, Pinto Ados S, Pinto LP, Cassano DS (2009). Maxillo-mandibular counter-clockwise rotation and mandibular advancement with TMJ Concepts total joint prostheses: part I—skeletal and dental stability. Int J Oral Maxillofac Surg.

[7] Wolford LM, Rodrigues DB, McPhillips A (2010). Management of the infected temporomandibular joint total joint prosthesis. J Oral Maxillofac Surg.

[8] Speculand B (2009). Current status of replacement of the temporomandibular joint in the United Kingdom. Br J Oral Maxillofac Surg.

[9] Saeed NR, McLeod NM, Hensher R (2001). Temporomandibular joint replacement in rheumatoid-induced disease. Br J Oral Maxillofac Surg.

[10] Movahed R, Mercuri LG (2015). Management of temporomandibular joint ankylosis. Oral Maxillofac Surg Clin North Am.

[11] Mercuri LG, Edibam NR, Giobbie-Hurder A (2007). Fourteen-year follow-up of a patient-fitted total temporomandibular joint reconstruction system. J Oral Maxillofac Surg.

[12] Westermark A (2010). Total reconstruction of the temporomandibular joint. Up to 8 years of follow-up of patients treated with Biomet® total joint prostheses. Int J Oral Maxillofac Surg.

[13] Giannakopoulos HE, Sinn DP, Quinn PD (2012). Biomet microfixation temporomandibular joint replacement system: a 3-year follow-up study of patients treated during 1995 to 2005. J Oral Maxillofac Surg.

